# MicroRNAs miR-29a-3p and miR-192-5p: promising urinary biomarkers for kidney function loss

**DOI:** 10.20945/2359-4292-2026-0019

**Published:** 2026-03-02

**Authors:** Cristine Dieter, Eliandra Girardi, Marcia Puñales, Daisy Crispim

**Affiliations:** 1 Serviço de Endocrinologia, Hospital de Clínicas de Porto Alegre, Porto Alegre, RG, Brasil; 2 Programa de Pós-graduação em Ciências Médicas: Endocrinologia, Faculdade de Medicina, Departamento de Clínica Médica, Universidade Federal do Rio Grande do Sul, Porto Alegre, RS, Brasil; 3 Programa de Pós-graduação em Saúde e Desenvolvimento Humano, Universidade La Salle, Canoas, RS, Brasil; 4 Instituto da Criança com Diabetes, Hospital Nossa Senhora da Conceição, Porto Alegre, RS, Brasil

**Keywords:** Diabetic kidney disease, microRNAs, miR-192-5p, miR-29a-3p

## Abstract

**Objective:**

This study aimed to evaluate the expression of miR-29a-3p and miR-192-5p in patients with type 1 diabetes mellitus (T1DM) with diabetic kidney disease (DKD) compared to those without DKD.

**Subjects and methods:**

This study included 29 patients with T1DM, comprising 13 without DKD (non-DKD group) and 16 with DKD, who were further subdivided into nine patients with moderate DKD and seven with severe DKD. MiR-29a-3p and miR-192-5p expression levels were measured in urine samples using qPCR and are presented as medians (25-75^th^ percentiles).

**Results:**

miR-29a-3p levels were higher in patients with DKD compared to the non-DKD group [1.24 (0.97-1.74) versus 0.83 (0.72-0.99); P = 0.008]. Its expression showed a negative correlation with estimated glomerular filtration rate (eGFR) (P = 0.007) and a positive correlation with creatinine levels (P = 0.004). MiR-192-5p levels were higher in patients with moderate DKD compared to the non-DKD group [2.15 (1.45-4.21) versus vs. 1.42 (0.98-2.45); P = 0.015], showing a negative correlation with eGFR (P = 0.003) and a positive correlation with creatinine (P = 0.006).

**Conclusion:**

The differential expression of miR-29a-3p and miR-192-5p in DKD highlights their potential as promising biomarkers for this complication.

## INTRODUCTION

Diabetic kidney disease (DKD) is a major chronic complication of diabetes mellitus (DM), affecting approximately 40% of individuals with DM and often progressing to end-stage renal disease ^(1)^. Key pathological features of DKD include glomerular mesangial expansion (hypertrophy), tubulointerstitial fibrosis, thickening of the glomerular basement membrane due to extracellular matrix (ECM) protein accumulation, and podocyte dysfunction, all of which collectively contribute to the development of proteinuria ^(1)^.

Early DKD detection is essential to prevent its progression to renal failure. Emerging research highlights the potential of novel biomarkers to identify the onset and progression of renal disease. Among these, microRNAs (miRNAs) stand out as a promising class of biomarkers. MiRNAs are small non-coding RNAs that regulate gene expression and are detectable in various human body fluids, including blood, saliva, and urine ^(2)^. They are critical in maintaining tissue homeostasis and driving disease processes, impacting nearly all fundamental cellular functions ^(3)^. Their stability in fluids and tissues is enhanced by protection from endogenous RNase activity, making them highly reliable for diagnostic purposes ^(2)^. Consequently, circulating miRNAs are increasingly recognized as valuable biomarkers for monitoring pathophysiological changes and predicting disease outcomes ^(2)^.

In this context, several studies have proposed different miRNAs as potential biomarkers for DKD ^(4,5)^. A systematic review by Assmann and cols. ^(6)^ highlighted that miR-21-5p, miR-29a-3p, miR-126-3p, miR-192-5p, miR-214-3p, and miR-342-3p are dysregulated in patients with DKD compared to the non-DKD group. Additionally, Lv and cols. ^(7)^ identified multiple miRNAs in plasma, urine, and exosomes as potential biomarkers for DKD. Among these, miR-29a-3p and miR-192-5p have garnered particular attention due to their involvement in DKD pathogenesis. Both miRNAs have been reported as dysregulated in DKD patients compared to controls; however, findings regarding their expression levels are inconsistent, with some studies reporting upregulation and others reporting downregulation [reviewed in ^(8)^]. For instance, Liu and cols. ^(9)^ reported an upregulation of miR-29a-3p in the serum of patients with type 2 DM (T2DM) compared to healthy controls. Interestingly, increased miR-29a-3p expression was associated with both the onset and progression of DKD ^(9)^. Conversely, miR-192-5p has been linked to a protective role in DKD, as its overexpression was shown to attenuate high-glucose-induced apoptosis and cell hypertrophy while improving the viability of proximal tubular cells ^(10)^.

Given the inconclusive findings on the roles of miR-29a-3p and miR-192-5p in DKD, this study aimed to investigate their expression levels in urine samples from patients with type 1 DM (T1DM) with and without DKD.

## SUBJECTS AND METHODS

### Study design and population

This study was conducted in accordance with the Strengthening the Reporting of Observational Studies in Epidemiology (STROBE) guidelines for association studies ^(11)^. It included an independent cohort of patients with T1DM stratified by estimated glomerular filtration rate (eGFR) values. All patients were recruited from outpatient clinics of Instituto da Criança com Diabetes - Grupo Hospitalar Conceição (Rio Grande do Sul State, Brazil), between November 2019 and December 2022. T1DM diagnosis was based on the American Diabetes Association criteria ^(12)^.

Patients were categorized into two groups based on their eGFR values: the normal eGFR group (≥ 90 mL/min/1.73 m^2^; non-DKD group) and the altered eGFR group (< 60 mL/min/1.73 m^2^; DKD group), in accordance with the Kidney Disease Improving Global Outcomes (KDIGO) guidelines ^(13)^. The DKD group was further subdivided according to the degree of kidney function loss: moderate DKD (moderate to severe loss, eGFR 30-60 mL/min/1.73 m^2)^ and severe DKD (severe loss leading to kidney failure, eGFR < 30 mL/min/1.73 m^2)^. The eGFR was calculated using serum creatinine levels and the Chronic Kidney Disease Epidemiology Collaboration (CKD-EPI) creatinine equation ^(13)^.

Exclusion criteria included any febrile illness within the previous three months, chronic inflammatory or rheumatic diseases, hepatitis, HIV positivity, glucocorticoid treatment, liver or cardiac failure, kidney transplantation, hereditary dyslipidemia, and inborn or acquired metabolic disorders, except for DM. This extensive list was carefully selected to minimize potential biases, as these conditions can affect miRNA expression. Moreover, all samples were collected in the morning to control for diurnal variations, which may also influence miRNA expression.

The study protocol was approved by the Research Ethics Committees of Hospital de Clínicas de Porto Alegre and Grupo Hospitalar Conceição/Instituto da Criança com Diabetes (HCPA No. 2019-0336, CAAE No. 13955019.0.0000.5327). All participants provided written informed consent prior to inclusion in the study.

### Clinical and biochemical parameters

A standardized questionnaire was used to collect information on age, age at diagnosis, T1DM duration, medication use, and ethnicity, which was determined by self-classification. All patients underwent physical and laboratory evaluations, as previously described ^(14)^. Serum creatinine was measured using the Jaffé reaction ^(15)^. The eGFR was calculated using the Chronic Kidney Disease Epidemiology Collaboration (CKD-EPI) equation: eGFR = 141 x min (SCR/κ, 1)^α^ x max (SCR/κ, 1)^-1,209^ x 0,993^age^ x 1,018 [if female] x 1,159 [if Black] ^(16)^.

### MiRNA extraction and quantification by qPCR

Midstream 20 mL voided urine samples were collected from all patients. Immediately after collection, samples were centrifuged at 3,500 x g for 5 min at 4 °C, and the supernatants were stored at -80 °C until miRNA expression analysis. Total RNA was extracted from 200 µL of urine using the miRNeasy Serum/Plasma kit (Qiagen; Hilden, Germany) following the manufacturer’s protocol. Quality control was performed using synthetic spike-in RNAs (RNA Spike-In Kit, Qiagen) to ensure the robustness of RNA isolation and the quality of extracted miRNAs. RNA isolation controls (UniSp2, UniSp4, and UniSp5; Qiagen) were included in thawed plasma samples before isolation to monitor extraction efficiency. Purity and concentration of RNA samples were assessed using the NanoDrop ND-1000 Spectrophotometer (Thermo Fisher Scientific, DE, USA).

After RNA isolation, 4 µL of total RNA was reverse transcribed into cDNA using the miRCURY LNA RT Kit (Qiagen), following the manufacturer’s instructions. The cDNA synthesis controls (UniSp6, Qiagen) and cel-miR-39-3p, provided in the RNA Spike-In Kit (Qiagen), were included in the reverse transcription reaction to assess the efficiency of the process.

Quantitative PCR (qPCR) was performed using a ViiA^TM^ 7 Fast Real-Time PCR System (Thermo Fisher Scientific) under the following cycling conditions: 95 °C for 2 min, followed by 40 cycles at 95 °C for 10 s and 56 °C for 1 min. Each sample was analyzed in triplicate, with a negative control included in every experiment. Relative quantification of hsa-miR-29a-3p (GeneGlobe assay ID: YP00204698) and hsa-miR-192-5p (GeneGlobe assay ID: YP00204099) was performed using the 2^-∆∆Cq^ method, with results expressed as fold change (FC) relative to a calibrator sample ^(17)^. MiR-93-5p (GeneGlobe assay ID: YP00204715) was used as the reference gene.

### Statistical analyses

The normality of variable distribution was assessed using the Kolmogorov-Smirnov and Shapiro-Wilk tests. Variables with a normal distribution are presented as mean ± standard deviation (SD), while those with a skewed distribution were log-transformed prior to analysis and are reported as median (25-75^th^ percentiles). Categorical data are expressed as percentages. Clinical and laboratory characteristics were compared between groups using one-way ANOVA for continuous variables or χ^2^ tests for categorical variables. Relative miRNA expression (qPCR data) was compared between groups using one-way ANOVA followed by post-hoc analysis. Correlations between quantitative variables were analyzed using Spearman’s correlation test. To investigate the discriminatory power of miRNAs in distinguishing DKD patients from T1DM controls, receiver-operating characteristic (ROC) curves were generated, and areas under the curves (AUCs) were calculated. All statistical analyses were conducted using SPSS software (version 18.0, SPSS Inc., Chicago, IL, USA), and P-values < 0.05 were considered statistically significant.

Using the OpenEpi web tool (https://www.openepi.com), we calculated that at least nine patients per group were required to achieve adequate statistical power (β = 80% and α = 0.05) to detect two fold change (± 1.5 SD) differences in miRNA expressions between groups. This sample size is consistent with previous studies analyzing miRNA expression ^(18-22)^.

## RESULTS

### Clinical and laboratory characteristics of individuals included in the study

**Table 1** summarizes clinical and laboratory characteristics of patients in the non-DKD and DKD groups. No significant differences were observed between groups regarding mean age, BMI, HbA1c, triglycerides, or cholesterol levels. Similarly, proportions of male and white participants did not differ significantly between patients with and without DKD (P > 0.500). As expected, the DKD group exhibited a higher prevalence of hypertension, elevated creatinine levels, and lower eGFR values compared to the non-DKD group (P < 0.050). Moreover, the prevalence of diabetic retinopathy (DR) was significantly higher among participants with DKD than in those without DKD (72.2% versus 16.7%; P = 0.003).

**Table 1:** Clinical and laboratory characteristics of patients included in this study

Characteristic	Non-DKD group (n = 13)	DKD group (n = 16)	P
Age (years)	32.3 ± 5.6	32.8 ± 5.2	0.784
Gender (% male)	50.0	44.4	1.000
BMI (kg/m^2)^	27.5 ± 4.2	26.4 ± 4.8	0.464
HbA1c (%)	7.8 ± 1.0	8.9 ± 2.4	0.110
Ethnicity (% of white)	83.3	72.2	0.688
Hypertension (%)	0.0	94.4	<0.001
Duration of T1DM (years)	21.7 ± 8.3	23.1 ± 7.3	0.598
Triglycerides (mg/dL)	85.0 (70.0 - 92.0)	109.0 (83.0 - 292.0)	0.121
Total cholesterol (mg/dL)	169.5 (141.0 - 182.3)	186.0 (157.0 - 203.0)	0.764
HDL cholesterol (mg/dL)	52.0 (47.0 - 71.7)	49.5 (36.2 - 61.7)	0.337
Creatinine (ug/dL)	0.8 (0.7 - 0.9)	2.4 (1.8 - 3.7)	<0.001
eGFR (mL/min per 1.73 m^2)^	114.3 ± 12.8	31.0 ± 13.6	<0.001
Diabetic retinopathy	16.7	72.2	0.003

Variables are shown as mean ± SD, median (25^th^-75^th^ percentiles) or %. BMI: body mass index; HbA1c: glycated hemoglobin; T1DM: type 1 diabetes mellitus.

### Expressions of hsa-miR-29a-3p and hsa-miR-192-5p in the urine of T1DM patients with and without DKD

**Figure 1:**
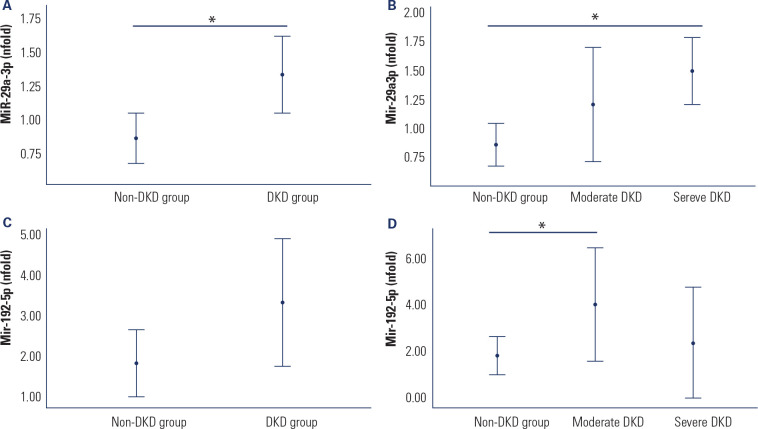
Mir-29a-3p and miR-192-5p expression levels in urine from T1DM patients with and without DKD. (**A**) Mir-29a-3p expression in the non-DKD and DKD groups. (**B**) Mir-29a-3p expression in patients classified as non-DKD, moderate DKD, and severe DKD groups. (**C**) MiR-192-5p expression in the non-DKD and DKD groups. (**D**) miR-192-5p expression in patients classified as non-DKD, moderate DKD, and severe DKD groups. Relative expression was quantified by qPCR. Data are shown as fold changes relative to the calibrator (∆∆Cq method) and are presented as median (25-75^th^ percentiles). P-values were obtained using ANOVA or Student’s *t*-tests, as applicable. *P < 0.050.

Expression levels of miR-29a-3p and miR-192-5p were evaluated in urine samples from T1DM patients, categorized according to the presence of DKD. As shown in **Figure 1A**, miR-29a-3p expression was significantly upregulated in patients with DKD compared to those in the non-DKD group [1.24 (0.97-1.74) versus 0.83 (0.72-0.99); P = 0.008]. When the DKD group was further stratified by the degree of kidney function loss, the highest miR-29a-3p levels were observed in patients with severe DKD compared to non-DKD patients [severe DKD: 1.42 (1.25-1.84); moderate DKD: 1.00 (0.78-1.59); non-DKD: 0.83 (0.72-0.99); P = 0.014; **Figure 1B**]. Similarly, miR-192-5p expression tended to be higher in DKD patients compared to the non-DKD group, although this difference did not reach statistical significance [2.14 (1.45-4.20) versus 1.42 (0.97-2.45); P = 0.084; **Figure 1C**]. Notably, this upregulation was more pronounced among patients with moderate DKD compared to non-DKD group [severe DKD: 1.77 (0.83-2.45); moderate DKD: 3.72 (1.66-4.61); non-DKD group: 1.42 (0.97-2.45); P = 0.043]; **Figure 1D**).

Next, we performed a correlation analysis between urinary mir-29a-3p and miR-192-5p expression levels and eGFR and creatinine values in all T1DM patients. Mir-29a-3p expression showed a negative correlation with eGFR (r = -0.490, P = 0.007) and a positive correlation with creatinine levels (r = -0.524, P = 0.004). In contrast, miR-192-5p expression did not show any significant correlation with DKD-related parameters (P > 0.050).

Moreover, we calculated the sensitivity and specificity of both miRNAs for detecting DKD using ROC curve analyses. For miR-29a-3p, the AUC was 0.945, with a sensitivity of 1.00 and a specificity of 0.923 in patients with severe DKD. The cut-off value for this miRNA was 1.144 to distinguish severe DKD patients from T1DM controls. Regarding miR-192-5p, the AUC was 0.812, with a sensitivity of 0.667 and a specificity of 0.846. The cut-off value was 2.955 to differentiate moderate DKD patients from T1DM controls.

## DISCUSSION

The identification of novel biomarkers for DKD is essential for detecting DM patients at risk of progressing to DKD or end-stage renal disease (ESRD). Among emerging candidates, miRNAs have been widely proposed as potential biomarkers for several diseases, including DKD. In this study, two miRNAs were highlighted as potential biomarkers for DKD: miR-29a-3p, which was associated with severe kidney function loss, and miR-192-5p, which demonstrated a significant association with moderate kidney function loss.

Here, miR-29a-3p expression was significantly upregulated in urine samples from T1DM patients with severe DKD compared to those without this complication. This finding aligns with Peng and cols. ^(23)^, who reported elevated urinary miR-29a-3p levels in patients with T2DM with albuminuria compared to those with normal urinary albumin excretion (UAE) levels. Additionally, miR-29a-3p levels were also positively correlated with UAE. Chien and cols. ^(24)^ similarly observed increased miR-29a-3p expression in plasma samples from patients with more advanced DKD compared to T2DM patients with normal UAE levels. Notably, higher plasma miR-29a-3p levels were associated with a rapid rise in creatinine, suggesting its potential as a prognostic marker for renal function decline in T2DM patients ^(24)^. A cross-sectional study performed by Liu and cols. ^(9)^ further demonstrated upregulation of miR-29a-3p in T2DM patients with DKD compared to controls. Consistent with our findings, miR-29a-3p expression was higher in the clinical proteinuria group (severe DKD) compared to the microalbuminuria group (moderate DKD) ^(9)^. The authors proposed serum miR-29a-3p as a potential diagnostic biomarker for DKD ^(9)^. Supporting these observations, miR-29a-3p was found to be upregulated in glomerular tissue from patients with DKD compared to those without this complication ^(25)^. Moreover, in patients with acute kidney injury (AKI), serum miR-29a-3p expression was significantly elevated compared to healthy controls, suggesting a potential association with AKI severity ^(26)^.

In contrast to our findings, Assmann and cols. ^(27)^ reported a downregulation of miR-29a-3p in plasma samples from T1DM patients with severe DKD compared to those with moderate DKD or without this complication. Similarly, Pezzolesi and cols. ^(28)^ found that miR-29a-3p was downregulated in the plasma of fast progressors to ESRD compared to non-progressors. These discrepancies may be explained by differences in sample types and methodological approaches. Our study analyzed urine samples, whereas Assmann and cols. ^(27)^ and Pezzolesi and cols. ^(28)^ focused on plasma samples. Additionally, DKD classification criteria varied across studies. In our analysis, DKD was defined solely on eGFR values, while Assmann and cols. ^(27)^ used a combination of eGFR and UAE levels, and Pezzolesi and cols. ^(28)^ used the urinary albumin-to-creatinine ratio along with eGFR.

The present study found that miR-192-5p expression was significantly upregulated in T1DM patients with moderate DKD compared to both those with severe DKD and the non-DKD group. Consistent with our findings, Jia and cols. ^(29)^ reported higher levels of miR-192-5p in urinary extracellular vesicles from T2DM patients with microalbuminuria compared to normoalbuminuric patients and healthy controls. They also observed a downregulation of miR-192-5p in patients with macroalbuminuria compared to those with microalbuminuria. Thus, their results also indicate that miR-192-5p is particularly elevated in patients with microalbuminuria. Additionally, Assmann and cols. ^(27)^ demonstrated an upregulation of miR-192-5p in plasma samples from T1DM patients with moderate DKD compared to both patients without DKD and those with severe DKD, further supporting its role as a biomarker for early renal impairment.

In contrast with these results, Akpinar and cols. ^(30)^ reported a downregulation of miR-192-5p in patients with DKD compared to controls. Their study included 50 healthy controls and 100 patients with T2DM, who were categorized into three subgroups based on UAE levels: normal to mildly increased (A1, n = 51), moderately increased (A2, n = 25), and severely increased (A3, n = 24) ^(30)^. Notably, the downregulation of miR-192-5p was most pronounced in patients in the A3 group. Similarly, Ma and cols. ^(31)^ observed significantly lower serum levels of miR-192-5p in T2DM patients with microalbuminuria compared to normoalbuminuric patients, with an additional decrease observed in those with macroalbuminuria relative to those with microalbuminuria.

In addition to their established roles in kidney injury, miR-29a and miR-192 are involved in systemic processes such as inflammation, oxidative stress, and extracellular matrix remodeling, contributing to cardiac fibrosis. These processes are key drivers of atherosclerosis development and progression, as well as left ventricular diastolic dysfunction, which are frequent comorbidities in patients with DKD ^(32)^. Additionally, other microRNAs, such as miR-423-5p, have been associated with glucose metabolism dysregulation and plaque progression in atherosclerosis ^(33)^. Given that many cases of chronic kidney disease result from diabetic nephropathy, these findings highlight multidirectional systemic consequences of altered microRNA expression. Future research could explore integrated biomarker panels that include miR-29a, miR-192, and other cardiovascular-related miRNAs to improve prediction of the interplay between renal and cardiovascular outcomes in diabetes.

Although most studies on DKD have been conducted in patients with T2DM, our analysis focused on individuals with T1DM, providing novel insights into the epigenetic mechanisms of DKD in this population. Since DKD development differs between diabetes types ^(34)^ - despite sharing some pathways - and factors such as obesity and dyslipidemia, more frequent in T2DM, can influence epigenetic profiles, studies targeting T1DM are particularly relevant. Another strength of this study is the use of urinary samples, which may better reflect kidney-specific processes compared to serum or plasma. A limitation of this study is the relatively small sample size; however, it is comparable to that used in most similar studies ^(20,21,35-40)^ and provided sufficient statistical power. Future studies with larger and longitudinal cohorts are warranted to validate and expand these findings.

The identification of novel biomarkers for DKD is essential for facilitating early detection and improving the management of patients at risk of disease progression. Our study demonstrates that miR-29a-3p and miR-192-5p are differentially expressed in patients with DKD, underscoring their potential as biomarkers for distinct stages of the disease. Their differential expression may serve as a valuable indicator of disease severity and progression, providing critical insights into the underlying pathophysiological mechanisms of DKD. These findings not only enhance our understanding of molecular pathways associated with DKD but also suggest promising avenues for incorporating these biomarkers into diagnostic and therapeutic strategies. However, further studies are required to validate these findings and explore their clinical applicability in larger and more diverse cohorts.

## Data Availability

all data generated or analyzed during this study are included in this published article.
